# Uterine Tissue Engineering and the Future of Uterus Transplantation

**DOI:** 10.1007/s10439-016-1776-2

**Published:** 2016-12-19

**Authors:** Mats Hellström, Sara Bandstein, Mats Brännström

**Affiliations:** 1 Laboratory for Transplantation and Regenerative Medicine, Department of Obstetrics and Gynecology, Sahlgrenska Academy, University of Gothenburg, Gothenburg, Sweden; 2 Kvinnokliniken, Blå stråket 6, 413 45 Göteborg, Sweden

**Keywords:** Bioengineering, Reproduction, Decellularization, Recellularization, Scaffold, Bioreactor, Mesenchymal stem cells

## Abstract

The recent successful births following live donor uterus transplantation are proof-of-concept that absolute uterine factor infertility is a treatable condition which affects several hundred thousand infertile women world-wide due to a dysfunctional uterus. This strategy also provides an alternative to gestational surrogate motherhood which is not practiced in most countries due to ethical, religious or legal reasons. The live donor surgery involved in uterus transplantation takes more than 10 h and is then followed by years of immunosuppressive medication to prevent uterine rejection. Immunosuppression is associated with significant adverse side effects, including nephrotoxicity, increased risk of serious infections, and diabetes. Thus, the development of alternative approaches to treat absolute uterine factor infertility would be desirable. This review discusses tissue engineering principles in general, but also details strategies on how to create a bioengineered uterus that could be used for transplantation, without risky donor surgery and any need for immunosuppression. We discuss scaffolds derived from decellularized organs/tissues which may be recellularized using various types of autologous somatic/stem cells, in particular for uterine tissue engineering. It further highlights the hurdles that lay ahead in developing an alternative to an allogeneic source for uterus transplantation.

## Introduction

### Uterus Transplantation

There has been great progress in the clinical field of solid organ transplantation during the last three decades, in particular since the introduction of calcineurin-inhibiting immunosuppressive drugs to effectively prevent graft rejection.^105^ These advancements have assisted clinical innovators to implement various types of vascularized composite tissue transplantation protocols for non-vital tissue transplantation such as the hand, forearm and face to enhance function and quality-of-life.^27^,^33^ After more than a decade of pre-clinical research on small- and large animal models,^12^,^24^,^28^,^34^,^48^,^78^,^79^,^109^^–^ 111 our group obtained enough skills and scientific evidence to receive ethical permission to evaluate uterus transplantation (UTx) as a mean to potentially cure absolute uterine factor infertility (AUFI), a condition of permanent infertility that is often associated with substantial socio-psychological discomfort.^93^ AUFI is caused by congenital or acquired uterine defects such as after severe intrauterine adhesions or due to partial uterine malformation, or after hysterectomy (due to cervical cancer, large leiomyoma or emergency peripartum hysterectomy) and affects about 1:500 women of fertile age.^67^

Using live donors, our group transplanted nine women who received a uterus to replace a uterus missing from birth (Mayer–Rokitansky–Küster–Hauser-syndrome)^59^ in all cases but one who lost her uterus after a hysterectomy as a treatment of early stage cervical cancer. Using standard anti-rejection treatment regimes, seven of these patients had graft survival for more than a year and were able to start embryo transfer attempts. Up until today, five healthy babies have been born and with further ongoing pregnancies.^10^,^11^,^13^,^14^,^70^ These successful outcomes demonstrated that UTx offers a curative treatment for infertility caused by AUFI. Several transplantation centers around the world are in the process of initiating clinical trials of UTx with some variations in techniques and donor source (with a deceased- or a live donor). Initial cases have just recently been reported in the media from China, USA and Czech Republic but have not yet been described in any peer-reviewed publications.

Allograft tissue is generally used for human transplantation procedures, including in a UTx setting. However, this is not used without complications. All transplanted patients face the risks of tissue rejection and immunosuppressant-related side effects.^85^ Long-term graft survival remains a challenge^5^,^9^ and patients become susceptible to infections and certain malignancies, including increased risks of hypertension, diabetes and accelerated arteriosclerosis.^5^ High doses of immunosuppressive drugs following UTx may, at least temporary, be required to halt progression of organ rejection. This thwarts potential embryo transfer attempts, which have been the case for one of the subjects in our clinical trial. Another issue using tissue matched donors for transplantation of life-depending organs is that the number of donors cannot be matched by the overwhelming number of people waiting for an organ transplant. A substantial waiting list mortality is therefore a great concern for patients in need of a new liver, kidney or a heart for example.^83^ The development of an alternative donor source, for example through the progress of an engineered grafting material based on the patient’s own cells to reduce rejection risks would greatly benefit the transplantation field, including for UTx patients. For these reasons, novel and promising concepts for functional organ- or tissue replacement have emerged within the fields of regenerative medicine and tissue engineering on a wide range of organs and tissues.^6^,^35^,^76^

### Basic Principles of Organ Tissue Engineering

Tissue engineering involves several steps, from the development of a template (or a scaffold), to tissue reconstruction using various cell sources. The scaffold could be either synthetic or biologically-derived and should serve to provide structural support for the added cells and aid cell proliferation and differentiation into an appropriate tissue specific cell fate. A normal organ may contain hundreds of millions, or even billions of cells. Thus the required cells need to be expanded to vast numbers and the engineered construct needs to be kept in an advanced perfusion bioreactors *in vitro* or to be grown ectopically *in vivo* to be finalized prior to its clinical application.

Scaffold generation has received much attention in the past years, in particular biological scaffolds since they, to a greater extent than synthetic scaffolds, mimic the native organ mechanically, geometrically and biologically.^76^ A biological scaffold can be obtained by a procedure called decellularization, a process where the immune provoking cells are removed from a normal organ which leaves a framework of tissue-specific three-dimensional (3D) extra cellular matrix (ECM).^6^ The ECM provides organ-specific tissue architecture with preserved vascular conduits, and contains important molecules, mainly type I collagen, glycosaminoglycans, fibronectin, laminin, elastin, and a diverse variety of growth factors with tissue specific composition.^23^ These molecules provide signals for cell aggregation, adhesion, migration, proliferation, and differentiation for that specific tissue.^23^,^47^,^76^,^81^ The ECM molecules are highly conserved between species, and as long as a balanced removal of hydrophilic and lipophilic antigens occur during decellularization, without a significant loss of ECM morphology, the decellularized scaffolds can avoid immune rejection in the recipient.^108^ These qualities make decellularized scaffolds interesting constructs for organ reconstruction studies.

Decellularization can be achieved by exposing the organ to either detergents, to ionic- or non-ionic solutions, to physical forces (such as freeze-thawing and high-/low pressure), or to various enzymatic treatments. Usually a combination of these methods is used. However, most are non-selective and will also damage ECM elements, particularly collagen, glycosaminoglycans, and resident growth factors.^36^ It is important to find a balance between an aggressive enough decellularization process that removes the immunogenic DNA and intracellular components while maintaining the ECM microenvironment intact, including patent conduits for the vasculature. With whole organs, the perfusion of decellularization chemicals through the vasculature is effective, but tissues can also be decellularized by immersing them in decellularization reagents. It is important to analyze the DNA content of the scaffolds throughout the process to make sure that sufficient DNA removal is accomplished to prevent rejection.^23^ Furthermore, washing the scaffold is crucial since remnants of the decellularization reagents may remain and cause adverse events during the reconstruction phase.

When an acellular scaffold has been created it may be implanted directly *in vivo* to recruit repopulating endogenous cells from the host, or (more commonly) cells can be integrated in the scaffold prior to implantation in a process generally termed “recellularization”. One major challenge in tissue engineering is to find an appropriate cell source for the recellularization of the scaffold. For whole-organ engineering, an ideal cell type is one that can proliferate and give rise to all cell types necessary for the particular organ to be regenerated, including the parenchyma, stroma, the vasculature, and all other supporting structures. For these reasons, many types of stem cells and progenitor cells have been evaluated, such as embryonic stem cells, induced pluripotent stem (IPS) cells, and various somatic stem cells. To date, embryonic- and mesenchymal stem cells are the most prevalent cell types used for recellularization.^86^ However, it is likely a mix of cell types that will be required for a successful reconstruction and future work will also focus on what sequence the different cell types should be applied in.

**Figure 1:**
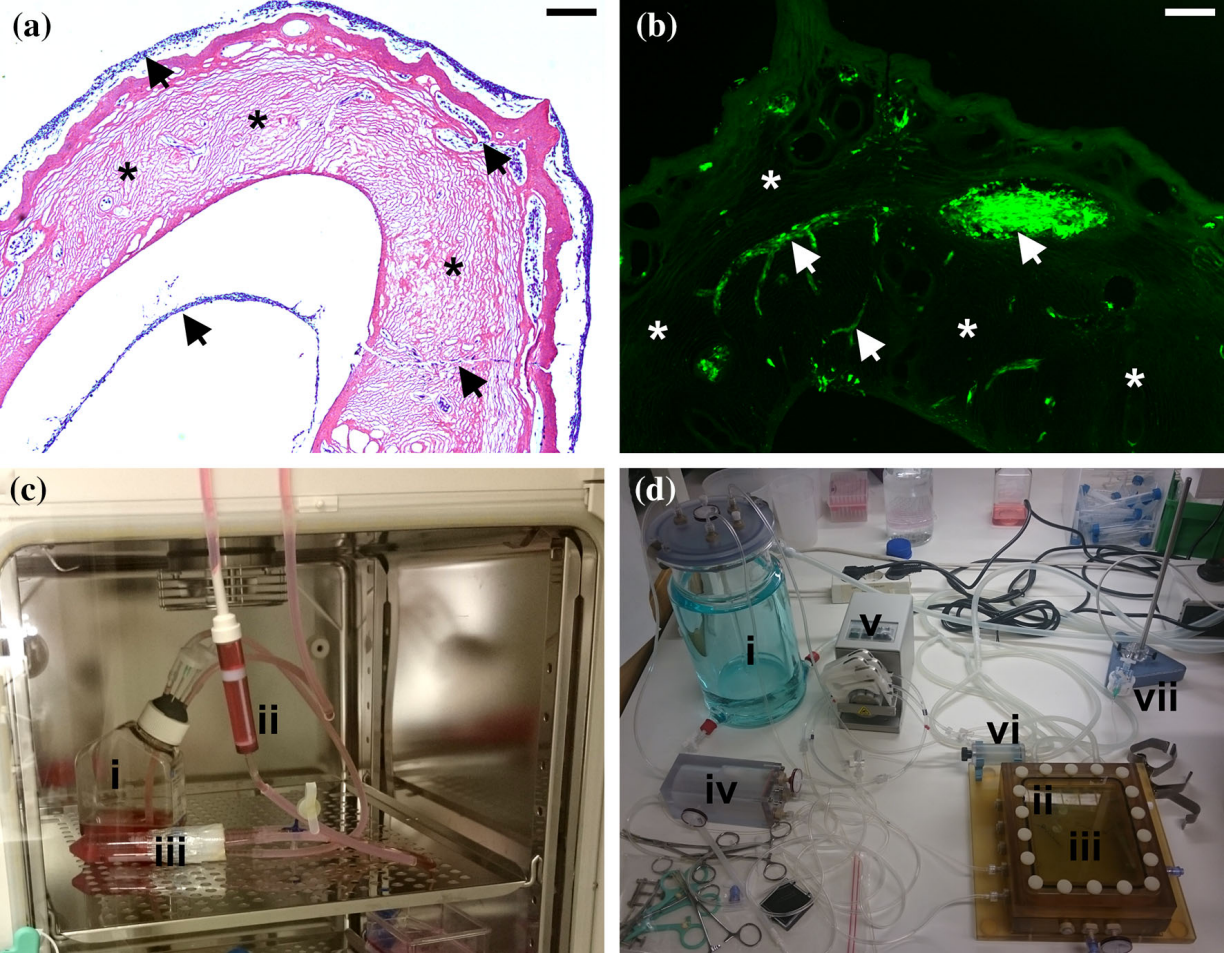
Previously unpublished pictures of a Hematoxylin and Eosin stained section (a), and of a section with green fluorescent protein (GFP) labeled cells (b) from a recellularized whole rat uterus scaffold that was kept for 7 days *in vitro* after recellularization with about 300 million rat GFP-labeled bone marrow derived mesenchymal stem cells (MSCs). The scaffold was generated by a decellularization protocol based on perfusing the ionic detergent sodium deoxycholate and deionized H_2_O sequentially for 5 days. After recellularization, the engineered uterus construct was kept submerged in media that circulated in a closed, homemade, perfusion bioreactor which was maintained in a 37 °C humidified chamber supplemented with 5% CO_2_ (c). Note the vast cell-free scaffold areas in (a, b), and that cells mainly localized around the outside and on the luminal side of the scaffold (a), and in isolated pockets within the scaffold (b). (c) Picture of the homemade bioreactor used for the particular experiment shown in (a, b). Note that this particular system did not provide any extra oxygen supply to the media. We have now invested in a highly sophisticated perfusion bioreactor system from Hugo Sachs Electronic—Harvard Apparatus GmbH (jacketed psu moist chamber with tubing heat exchanger type 834/10) which gives us much better conditions for 3D-cell culturing, including temperature regulation, media oxygenation and pressure measurements (d). All animal experiments were approved by the Animal Ethics Committee in Gothenburg, Sweden. We are currently optimizing our recellularization techniques using this innovative system which hopefully also will extend our culturing times and reduce the contamination prevalence which has been a significant problem. Scale bar 200 *µ*m. i, media cistern; ii, bubble trap; iii, organ perfusion site and reservoir; iv, oxygenator; v, peristaltic pump (not shown in c); vi, media heat exchanger; vii, pressure measure device.

A successful recellularization also requires optimal cell delivery methods and culturing conditions. The two main methods for cell delivery are either perfusion of cells through the vasculature, or by repeated injections of cells into the scaffold using a syringe. Perfusion would be the choice in order to reach the vasculature, whereas injections of cells target the parenchyma and stroma more directly. Thus, a combination of the two has been the approach by many groups, including us (Figs. 1a and 1b). The culturing conditions also matters on the recellularization efficiency, and one of the advantages with using decellularized biological tissues is that the vascular conduits are preserved. This route is therefore commonly used to cannulate and connect to various custom made- or commercially available perfusion bioreactors (Figs. 1c and 1d). Negative pressure in the culturing chamber may also be used to mechanically enhance cell migration and seeding efficiencies of decellularized organs.^92^

### Progress in Organ Tissue Engineering Using Decellularized Matrixes

The field of organ tissue engineering is still in its infancy and many challenges remain before the development of complex organs will be established. Modest steps have been made in small animal organ bioengineering, where rudimentary *in vivo* function and patency were observed for a very limited time.^73^,^74^,^77^,^92^,^100^ For example, tissue engineered rat livers showed maintained hepatocyte viability and to some extent metabolic function by producing liver-specific proteins *in vitro* and *in vivo.* 7,55,100 *In vitro* results of macroscopic contractions and pump function of a decellularized rat heart was also established, describing that cardiovascular progenitor muscle cells and endothelial cells could migrate, proliferate and differentiate into cardiomyocytes, smooth muscle cells and endothelial cells.^74^ Furthermore, human umbilical vein endothelial cells and rat neonatal kidney cells were used to recellularize rat kidney scaffolds that developed into specialized kidney cells.^92^

The creation of bioengineered tissue parts is less complicated than the creation of complete organs and have in some areas successfully been clinically applied using biologically- or synthetically derived scaffolds; for example engineered skin-,^4^,^69^ bone-94 and heart valve grafts.^50^ Researchers have also clinically tested bioengineered constructs of “less complex” hollow structures such as urogenital tissues,^3^ blood vessels 57,71 and trachea.^39^,^49^,^64^ Due to raised concerns, a few studies are currently being re-evaluated by external scientific- and ethical organizations.^53^ Nevertheless, many tissue engineering studies show great promise, evidenced by the vast number of pre-clinical reports using decellularization- and recellularization methods for many different organs and tissues.^16^,^38^,^44^,^46^,^58^,^63^,^95^,^103^

### Tissue Engineering the Reproductive Organs

Several sites of tissue engineering of female reproductive organs have been investigated,^2^,^82^,^89^ including the lower female reproductive tract.^25^,^80^,^97^,^98^ For fertility aid, ovarian- and follicle-related bioengineering applications have particularly been explored as means to cure infertility caused by conventional anti-cancer treatments.^89^ Fertility is particularly challenging to retain in leukemia-treated females where the otherwise successful method of re-transplanting cryopreserved ovarian tissue is unsafe due to the risk of reintroducing malignant cells.^30^^–^ 32 However, there are many pre-clinical reports describing various successful bioengineered supporting structures for isolated early-stage follicles which were able to support *in vitro* follicle maturation.^40^,^61^,^62^,^90^,^91^,^101^,^102^,^107^,^112^ These are significant achievements since the 3D microstructure used to encapsulate growing follicles *in vitro* must be plastic enough to allow a massive exponential growth in volume (in particular for large mammals) and yet also provide crucial physical support for oocyte–somatic cell connections that promote follicular development throughout the whole process.^89^ Collectively, these studies provide hope to the development of future safe methods to preserve fertility for leukemia treated patients.

## Uterine Tissue Engineering

Engineered 3D uterine tissue culturing systems have been used for a number of years to perform decidual differentiation- and embryo implantation studies.^51^,^66^,^88^ The majority of published work was focused on fertility issues, however tissue engineered constructs were also used as *in vitro* models to study invasion mechanisms of endometrial cancer cells^8^,^75^ and epithelial- and stromal cell communication.^1^ Several studies used scaffolds derived from collagen.^29^,^60^,^87^,^104^ For example, Lu *et al*. created a uterus-like stratified construct *in vitro* by sequentially adding rabbit myometrial-, endometrial-, and epithelial cells in a collagen/matrigel mix on top of an agar bed. These constructs supported mouse embryo development and maintained their quality better than the control group (mouse embryos grown in normal cell culture flasks using the same media).^60^ In a similar study, the reconstructed rabbit endometrium was improved when using estrogen/progesterone stimulated endometrial cells.^104^ Collagen-coated silk scaffolds have been used to construct human cervix-like tissue *in vitro* and results showed that a dynamic cell culturing system with human cervix cells isolated from the cervical stroma (mid canal region) significantly enhanced the concentration of ECM-related molecules and scaffold stiffness when spinner flasks were used over an extended time period of eight weeks.^45^ In another study, bone marrow derived mesenchymal stem cells (BM-MSCs) were cultured on collagen scaffolds which were then tested *in vivo* to repair a full-thickness uterine wall injury in a rat model.^29^ Evaluated at 30- and 90 days after transplantation, grafts improved the healing process in the uterine wall by inducing proliferative abilities of host uterine endometrial- and muscular cells and facilitated microvasculature regeneration. Embryo development also took place within the grafted area, although authors do not discuss if the actual placentation was formed over the grafts (which would have proved optimal reconstruction and vascularization of the engineered tissue).^29^

An earlier report describes myofibroblast-encapsulated grafts that were created by transplanting boiled blood cloths molded into tubular shapes (5 × 25 mm in size) into the peritoneal cavity.^18^ The peritoneal cavity was used as a host myofibroblast recruitment site and as a kind of *in vivo* bioreactor. Two- to three weeks after initial surgery, the myofibroblast-encapsulated grafts were harvested and the tubular shaped blood cloths were carefully removed so that the myofibroblast (now also tubular shaped) tissue could be isolated. This myofibroblast tissue was then used to repair an injury in the uterine wall of the same animal. The grafts subsequently became more uterine-like over the following twelve weeks. Evident structural complexity was observed, including columnar epithelium, secretory glands, and muscle bundles which were organized into two distinct layers, but with some organizational differences compared to normal uterine tissue. In the same study, pregnancies that were induced 12 weeks post-transplantation showed that grafts could physically support a growing uterus up to embryonic day (E) 20. It is somewhat unclear, but to our understanding, placentation did not appear to have happened in the grafted tissue itself, but rather in other areas of normal uterine tissue within the grafted horn.^18^ This study does not only show the regenerative capacity of the rat uterus, but also describe an innovative technique on how to use the peritoneal cavity of the host as a bioreactor, which perhaps should be explored further with other constructs.

In two published review papers, a uterine tissue transplantation study has been reported using the rabbit model.^2^,^82^ Pre-configured uterine-shaped biodegradable polymer scaffolds seeded with autologous uterine smooth muscle cells and epithelial cells were used to construct bioengineered uterine tissue for a “subtotal uterine tissue replacement” in the corresponding autologous animal. Based on unpublished results from immunohistochemistry, western blot and biomechanical analyzes using organ bath assays of the grafts, they reported normal uterine tissue components with functional characteristics similar to those of normal uterine tissue.^82^

To our knowledge, it is not until recently that uterine bioengineering studies have investigated the use of decellularized material as scaffolds to reconstruct uterine tissues. Pregnant rat- and human myometrium specimen were decellularized using an ethanol/water/trypsin decellularization process, which initially was optimized for large arteries.^113^ These scaffolds were then recellularized with various human and rat myocyte cell lines to be evaluated *in vitro*. Interestingly, the human cells adapted better to the decellularized rat scaffolds. These constructs were cultured for up to 51 days *in vitro* which gave the cells enough time to form multicellular layers on the scaffold surface and clusters of cells within the depths of the rat scaffold. These constructs showed some contractility in an organ bath, indicating elementary uterus phenotype-like functionality.^113^ However, no repopulation of the vascular conduits was observed which may have affected the cell homeostasis deeper in the tissue layers. Santoso *et al*. also investigated decellularized uterine tissue segments.^84^ They compared three different decellularization methods for full-thickness rat uterine segments, using protocols based on (1) sodium dodecyl sulfate (SDS), (2) Triton-X100, and (3) high hydrostatic pressure (HHP). After detailed analysis, the authors concluded that SDS and HHP were more effective to obtain decellularization than Triton-X100. When cell-free SDS- and HHP-derived scaffold segments were transplanted to repair a defect uterine wall and analyzed four weeks later, it was evident that both scaffold types supported local recruitment of host uterine cells which were positive for estrogen receptor markers. Pregnancy tests made on the operated rats, also suggested that the constructs gave some level of structural support during fetal development.^84^ In a recent study, a similar experimental approach was used in the mouse model where cell-free decellularized mouse uterus segments were transplanted to ovariectomized mice or to Stat3 conditional knockout mice.^43^ The results showed that the spontaneous host uterus regeneration process was independent of ovarian hormones but highlighted a key role for Stat3 in the regeneration process. Collectively, these studies provide encouraging results for various bioengineering strategies for partial uterine repair which is clinically relevant in a situation to cover partial uterine defects caused for example by resection of placental tumors, extensive myomectomy or adenomyomectomy. A tissue engineered uterine patch may in these cases be used to increase the strength of the uterine wall in a future situation of pregnancy.

However, concerning whole-uterus bioengineering applications, larger constructs with an appropriate vasculature that can be anastomosed to the host vasculature will be necessary. For these reasons, and in line with what was developed for other whole organs,^73^,^74^,^77^,^92^,^100^ we and one other group evaluated different strategies to decellularize whole rat uterus. The rat uterus was isolated with an intact vascular tree (from the aorta to vena cava) from a donor rat so that a whole uterus scaffold could be obtained by decellularization, which then enable recellularization and transplantation with vascular anastomoses.^41^,^68^ Miyazaki and Maruyama used an SDS-based decellularization protocol originally optimized for the rat liver.^100^ At the same time, we developed three different decellularization protocols; two protocols were based on a combination of Triton-X100 and dimethyl sulfoxide (DMSO), where one protocol was buffered in phosphate buffered saline (PBS) for the duration of the procedure, while the other was non-buffered (de-ionized H_2_O).^41^ Our third protocol was based on an ionic detergent called sodium deoxycholate (SDC).^41^ For all three protocols, the detergents and ionic solutions were alternately perfused through the rat uterus via the vascular system for a total of 5 days. During this process, the cell membranes were targeted by the detergents, while the osmotic alternations assisted cell lysis. DNA and other cell remnants where subsequently washed away.^41^ Together, our two separate studies provide an excellent platform on to which novel whole-uterus tissue engineering experiments can be developed from. However, the treatment differences during scaffold generation changes the physical, mechanical and biomolecular properties of the uterine scaffolds, which potentially impact their functionality. Thus, before progressing further on whole-uterus bioengineering applications, or to larger animal models, we should try to establish which scaffold design is the most suitable for the cellular reconstruction and the successive *in vivo* applications. For these reasons, Miyazaki and Maruyama and Hellström *et al*. performed similar patch transplantation studies as to what Ding *et al*. and Santoso *et al*. did.^29^,^42^,^68^,^84^ Miyazaki and Maruyama preconditioned their SDS-derived whole-uterine scaffolds with collagen, which were then recellularized by multiple injections with a total of about eighty million cells per scaffold from a cell mix of neonatal- and adult uterine cells and BM-MSCs (with a ratio of about 51:27:1, respectively). Ten days after recellularization and culture in a perfusion bioreactor, segments were cut out (15 × 5 mm) from the recellularized whole-uterus scaffold and transplanted to replace a full-thickness uterine wall segment of the same size by continuous 6-0 prolene sutures. Twenty-eight days following engraftment, immunohistochemistry showed a large number of cells positive for vimentin (stromal cells), cytokeratin (epithelial cells) and smooth muscle actin (myometrium). Pregnancies were achieved and fetal weights were normal in the operated horns, but the total number of fetuses was significantly reduced compared to normal pregnant animals and no placentation had formed over the graft. Perhaps these results could have been improved further if the pregnancy was induced at a later time point after surgery. Indeed the cell quantity and organization were improved ninety days following engraftment.^68^

In collaboration with Dr. Miyazaki and Dr. Maruyama, we recellularized segments (20 × 5 mm) of decellularized uterus tissue by injecting about 7 million GFP-labeled BM-MSCs (GFP-BM-MSCs) and adult primary uterine cells (ratio of 150:1, respectively). Three days after recellularization, the constructs were cut in half so that one half could be analyzed and the other half transplanted to repair a full-thickness uterine wall defect (10 × 5 mm).^42^ Sections of the constructs analyzed histologically prior to transplantation showed that very few cells were dispersed into the deeper layers of the scaffolds. Except for some cell clusters in isolated pockets within the scaffolds, the cells generally remained on the surfaces of the scaffold.^42^ Since very few cells were GFP negative, we used fluorescence, confocal microscopy and automated software to quantify the cell confluency on the scaffold surfaces. It turned out that the scaffolds produced with the mild Triton-X100/DMSO treatment were better at supporting the recellularized cells compared to SDC-derived scaffolds, in particular using the buffered protocol.^42^ The buffered decellularization protocol generated uterus scaffolds with significantly higher amounts of sulfated glycosaminoglycans (sGAGs; 2.8–3.7 times higher compared to the non-buffered protocols) which may have led to the improved recellularization.^41^ Using proteomics, we also identified higher levels of certain collagens and proteoglycans (i.e. biglycan, decorin, lumican) and a basal lamina-related protein (nidogen-2) in the Triton-X100/DMSO-generated scaffolds.^41^ Scaffold porosity and stiffness also play an important role in how stem cells behave.^54^,^96^,^106^ Our SDC-derived scaffolds were 1.5–1.7 times stiffer and more porous than our Triton-X100/DMSO-derived scaffolds which showed a very fiber-rich, but compact substrate.^41^ It can be speculated that these physical differences also played an important role in the recelluarization outcomes. However, *in vitro* migration and proliferation were generally poor in all scaffolds,^42^ thus culturing conditions (and perhaps scaffold generation) need further optimization (Table 1).

**Table 1:** A summary of the uterus tissue engineering studies mentioned in the text.

Uterine tissue investigated	Species	Scaffold material	Cells used	Time *in vitro*	Culturing conditions	Transplanted *in vivo*?	Size of graft	Pregnancy tests?	References
Endometrium	Human	Collagen/matrigel	Human endometrium cells	10 days	Static culture, modified alpha-medium	No	N/A	N/A	Meng *et al*.^66^
Endometrium	Human	Collagen/matrigel	Human endometrial epithelial with stromal cells	48 h	Static culture, serum-free DMEM/F-12 medium with steroids/hormones	No	N/A	N/A	Kim *et al*.^51^
Endometrium	Human	Collagen biomatrix	Human endometrial cells	3 days	Static culture, serum-free DMEM/F-12 medium with steroids/hormones	No	N/A	N/A	Sengupta *et al*.^88^
Endometrium	Human	Collagen/matrigel	Human endometrial stromal cells and epithelial cells	3–5 days	Static culture, DMEM/F12 with high glucose plus 10% FBS with steroids/hormones	No	N/A	N/A	Park *et al*.^75^
Endometrial carcinogenetic tissue	Human	Collagen	Human endometrial carcinogenetic cells	2 weeks	Static culture, modified MEM media	No	N/A	N/A	Benbrook *et al*.^8^
Endometrium	Human	Matrigel	Human endometrial epithelial with stromal cells	1–2 weeks	Static culture, M199/F12 media with serum and steroids/hormones	No	N/A	N/A	Arnold *et al*.^1^
Full thickness uterine tissue	Rat	Collagen	Bone marrow-derived mesenchymal stem cells (BM-MSCs)	72 h	Static culture, LG-DMEM containing 12.5% fetal bovine serum and steroids/hormones	Yes, for 105–109 days	15 × 5 mm	Yes, 90 days post transplantation	Ding *et al*.^29^
Full thickness uterine tissue	Rabbit	Collagen/matrigel	Epithelial, stromal and smooth muscle cells from rabbit uterus and mouse embryos	14–16 days	Static culture, DMEM/F12 medium + 10% FBS	No	N/A	N/A	Lu *et al*.^60^
Endometrium	Human	collagen hydrogel	Telomerase immortalized human endometrial stromal cells	10–12 days	Static culture, DMEM/F12 + 10% FBS with steroids/hormones	No	N/A	N/A	Schutte *et al*.^87^
Endometrium	Rabbit	Collagen/matrigel	Rabbit endometrial cells and mouse embryos	14 days	Static culture, DMEM/F12 + 20% FBS with steroids/hormones	No	N/A	N/A	Wang *et al*.^104^
Cervix tissue	Human	Collagen/silk	Human cervical cells (from the mid canal region	4–7 weeks	spinner flask system, DMEM + 10% FBS	No	N/A	N/A	House *et al*.^45^
Full thickness uterine tissue	Rat	Myofibroblast-rich tissue, shaped with aid from boiled blood cloths	Rat myofibroblasts	*In vivo*, 2 weeks	In the peritoneal cavity	Yes, for 4–12 weeks	15–20 × 7.5–10 mm	Yes, at 4, 6 and 12 weeks post transplantation	Campbell *et al*.^18^
Myometrium	Rat Human	Decellularized rat and human myometrial segments (ethanol/trypsin based protocol)	Human and rat myocytes	Up to 51 days	Static culture, DMEM with 10% fetal bovine serum	No	N/A	N/A	Young *et al*.^113^
Full thickness uterine tissue	Rat	Decellularized rat uterus segments (SDS/Triton X-100/High hydrostatic pressure based protocols)	None	N/A	N/A	Yes, for up to 51 days	15 × 5 mm	Yes, 30 days post transplantation	Santoso *et al*.^84^
Full thickness uterine tissue	Mouse	Decellularized mouse uterus segments (SDS based protocols)	None	N/A	N/A	Yes, for up to 7 weeks	5 × 2 mm	Yes, 4 weeks post transplantation	Hiraoka *et al*.^43^
Full thickness uterine tissue	Rat	Decellularized whole rat uterus^59^ or segments^70^ (Triton X-100 + DMSO or SDC based protocols)	Rat GFP-labeled BM-MSCs and primary uterus cells	3 days	Static culture, DMEM with 10% fetal bovine serum	Yes, for 9 weeks	10 × 5 mm	Yes, 6 weeks post transplantation	Hellström *et al*.^41^ and Hellstrom *et al*.^42^
Full thickness uterine tissue	Rat	Decellularized whole rat uterus (SDS based protocol)	Rat neonatal, adult uterine cells and rat BM-MSCs	Up to 10 days	Perfusion Bioreactor, Smooth Muscle Cell Basal Medium 2 with 5% FBS with steroids/hormones	Yes, for up to 90 days	15 × 5 mm	Yes, 28 days post transplantation	Miyazaki and Maruyama^68^

The recellularized triton-X100/DMSO-derived scaffolds also worked better *in vivo* compared to the SDC-derived constructs; grafts showed a higher degree of homing affect and supported a spontaneous host cell infiltration and organization better. As evidenced by immunohistochemistry and qPCR, all cells used in the recellularization process were replaced by infiltrating host cells in all constructs. However, the GPF-MSCs contributed to the spontaneous host cell reconstruction of the grafted tissue since we noticed that cell free, acellular, scaffolds had completely degraded 3 months after transplantation *in vivo.* 42 We obtained similar numbers of fetuses in the operated horns compared to the non-operated horn when using constructs derived from the Triton-X100/DMSO-produced scaffolds. To our knowledge, these pregnancy results are better than other published uterine tissue engineering studies. We considered it important to analyze the construct in detail prior to transplantation, which therefore reduced our grafting size material to become 5 mm smaller in size than that of other studies, and this fact may have influenced our *in vivo* results. Consequently the results are not easily comparable to other studies using larger grafts. Other differences between the studies include the various time points when pregnancy was induced. For example, we waited 42 days after transplantation (6 weeks), Miyazaki and Mauyama waited 28 days (4 weeks), Ding *et al*. waited 90 days and Santoso *et al*. waited 30 days.^29^,^42^,^68^,^84^ Naturally this has an influence on the fertility outcomes in the operated horns since construct maturity generally seem to improve over time after transplantation. Other protocol- and technical variations between research groups make it difficult to directly compare construct functionality based solely on fetal numbers. In our latest study, fetal development occurred in the grafted area in two animals in both Triton-X100/DMSO groups, showing that the uterine wall containing the graft was strong enough to support a near full-term pregnancy (E16–E20).^42^ However, we also did not see any placentation formed directly over the engineered graft itself. The reason behind this is unclear; we speculate that this may be due to an insufficient blood supply to the graft since the transplantation procedure does not allow for any vascular anastomoses. Nevertheless, an insufficient cell number, tissue organization and/or poor epithelium reconstruction may also be accountable. Many areas of the grafts showed near-uterus like morphology with well-developed myometrial-like features and a uniformed and lined epithelial layer. However, we also noticed a possible immune cell infiltration, granulation formation and angiogenesis to other areas of the grafts.^42^ It is unclear whether this response was caused by the scaffold, the GFP-labeled cells, or the fact that we used an outbred rat strain for the experiment. We also do not know if these events are detrimental or beneficial since immune system activation can favor tissue regeneration under particular circumstances.^15^,^22^,^65^ A favorable immune response involves alternatively-activated (M2) macrophages, which contribute to anti-inflammatory, angiogenic and tissue remodeling responses. Conversely, the classically-activated (M1) macrophages have been linked to inflammation, tissue destruction, microbial destruction and clearance of apoptotic cells.^22^ There is limited information on the immunological responses following uterine tissue engineering transplantations, and these important issues need to be addressed. Especially, since one of the key objectives is to develop an optimal patient-specific grafting material to avoid the use of immunosuppressive drugs. However, a temporary immunosuppressive treatment-regime may be favorable to suppress a possible detrimental M1-related immune response, and to instead potentiate a beneficial M2-related stimulus after transplantation. Indeed, a higher ratio of M2 macrophages vs. M1 macrophages was associated with better transplantation and remodeling outcomes of other non-uterus related decellularized materials in a rat model.^17^ Transplanted BM-MSCs have been suggested to act in an immune modulating manner through paracrine actions in many systems,^52^,^56^,^65^ including the uterus^20^,^21^ and therefore seem to have an important role to play in tissue engineering.

The choice of cells used for the recellularization process plays a major role in the outcome of the tissue reconstruction. We believe that it is likely that a mix of various cell types, including endothelial cells, will be required to reconstruct a functional uterine tissue from decellularized tissues. Uterus side population cells^19^,^21^,^72^ or endometrial mesenchymal stem/stromal cells^26^,^37^,^99^ have shown somatic uterine stem cell characteristics and an ability to reconstruct uterine tissue. These cells may therefore be suitable cell sources for uterine tissue engineering applications. However, for women who completely lack uterine tissue, other autologous cells sources such as BM-MSCs and/or IPS cells should be explored.

## Concluding Remarks and Hurdles to Overcome

Great progress has been made in the last decade in regenerative medicine, including how to bioengineer uterine tissues. Several promising scaffold designs have been established but a major limitation is the *in vitro* and *in vivo* recellularization efficiency. This matter may be caused by an insufficient scaffold homeostasis and/or by inadequate *in vitro* 3D culturing conditions, which can affect the balance between hypoxia, repopulation and re-vascularization of the construct. The outcome from these factors may also be cell type-dependent; hence, more studies focusing on the culturing conditions and the various cell sources, including uterine- and endothelial-associated cell types, IPS-cells, and/or various somatic stem cells added in a specific sequence during the reconstruction process should be explored in future research. However, vast expansion of pluripotent cells may lead to undesirable phenotypic changes which also need to be considered. The immune response following engineered uterine tissue transplantation should also be deeper investigated since it plays a significant role in the regeneration- or the destruction of the grafts. However, the construction of bioengineered uterine patches, which may be clinically applied to repair a partial defect in the uterine wall, have come a long way and it may be time to move forward and evaluate some of these constructs in larger animal models. Results from such experiments would certainly contribute to our understanding on how to construct a whole bioengineered uterus that can replace a donor in a UTx setting, which still is in its initial stages of development.

## Abbreviations

AUFI: Absolute uterine factor infertility
BM: Bone marrow
DMSO: Dimethyl sulfoxide
E: Embryonic day
ECM: Extra cellular matrix
GAGs: Glycosaminoglycans
GFP: Green fluorescent protein
HHP: High hydrostatic pressure
IPS: Induced pluripotent stem
M1: Classically-activated macrophages
M2: Alternatively-activated macrophages
MSCs: Mesenchymal stem cells
PBS: Phosphate buffered saline
SDC: Sodium deoxycholate
SDS: Sodium dodecyl sulfate
sGAGs: Sulphated glycosaminoglycans
UTx: Uterus transplantation

## References

[1] Arnold JT, Kaufman DG, Seppala M, Lessey BA (2001). Endometrial stromal cells regulate epithelial cell growth in vitro: a new co-culture model. Hum. Reprod..

[2] Atala A (2012). Tissue engineering of reproductive tissues and organs. Fertil. Steril..

[3] Atala A, Bauer SB, Soker S, Yoo JJ, Retik AB (2006). Tissue-engineered autologous bladders for patients needing cystoplasty. Lancet.

[4] Atiyeh BS, Costagliola M (2007). Cultured epithelial autograft (CEA) in burn treatment: three decades later. Burns.

[5] Azimzadeh AM, Lees JR, Ding Y, Bromberg JS (2011). Immunobiology of transplantation: impact on targets for large and small molecules. Clin. Pharmacol. Ther..

[6] Badylak SF, Taylor D, Uygun K (2011). Whole-organ tissue engineering: decellularization and recellularization of three-dimensional matrix scaffolds. Annu. Rev. Biomed. Eng..

[7] Baptista PM, Siddiqui MM, Lozier G, Rodriguez SR, Atala A, Soker S (2011). The use of whole organ decellularization for the generation of a vascularized liver organoid. Hepatology.

[8] Benbrook DM, Lightfoot S, Ranger-Moore J, Liu T, Chengedza S (2008). Gene expression analysis of biological systems driving an organotypic model of endometrial carcinogenesis and chemoprevention. Gene Regul. Syst. Biol..

[9] Bluestone JA, Auchincloss H, Nepom GT, Rotrosen D, St Clair EW, Turka LA (2010). The immune tolerance network at 10 years: tolerance research at the bedside. Nat. Rev. Immunol..

[10] Bokström, H., P. Dahm-Kahler, H. Hagberg, L. Nilsson, M. Olausson, and M. Brännström. Livmodertransplantation i Sverige—5 första barnen i världen födda—Lovande resultat—alla barn friska. *Lakartidningen* 113, 2016. 27003530

[11] Brännström M, Bokström H, Dahm-Kahler P, Diaz-Garcia C, Ekberg J (2016). One uterus bridging three generations: first live birth after mother-to-daughter uterus transplantation. Fertil. Steril..

[12] Brännström M, Diaz-Garcia C, Hanafy A, Olausson M, Tzakis A (2012). Uterus transplantation: animal research and human possibilities. Fertil. Steril..

[13] Brännström M, Johannesson L, Bokstrom H, Kvarnström N, Molne J (2015). Livebirth after uterus transplantation. Lancet.

[14] Brännström M, Johannesson L, Dahm-Kahler P, Enskog A, Mölne J (2014). The first clinical uterus transplantation trial: a six-month report. Fertil. Steril..

[15] Brennan FH, Anderson AJ, Taylor SM, Woodruff TM, Ruitenberg MJ (2012). Complement activation in the injured central nervous system: another dual-edged sword?. J. Neuroinflamm..

[16] Brown AL, Brook-Allred TT, Waddell JE, White J, Werkmeister JA (2005). Bladder acellular matrix as a substrate for studying in vitro bladder smooth muscle-urothelial cell interactions. Biomaterials.

[17] Brown BN, Londono R, Tottey S, Zhang L, Kukla KA (2012). Macrophage phenotype as a predictor of constructive remodeling following the implantation of biologically derived surgical mesh materials. Acta Biomater..

[18] Campbell GR, Turnbull G, Xiang L, Haines M, Armstrong S (2008). The peritoneal cavity as a bioreactor for tissue engineering visceral organs: bladder, uterus and vas deferens. J. Tissue Eng. Regen. Med..

[19] Cervello I, Gil-Sanchis C, Mas A, Delgado-Rosas F, Martinez-Conejero JA (2010). Human endometrial side population cells exhibit genotypic, phenotypic and functional features of somatic stem cells. PloS ONE.

[20] Cervello I, Gil-Sanchis C, Santamaria X, Cabanillas S, Diaz A (2015). Human CD133(+) bone marrow-derived stem cells promote endometrial proliferation in a murine model of Asherman syndrome. Fertil. Steril..

[21] Cervello I, Santamaria X, Miyazaki K, Maruyama T, Simon C (2015). Cell therapy and tissue engineering from and toward the uterus. Sem. Reprod. Med..

[22] Chamberlain MD, West ME, Lam GC, Sefton MV (2015). In vivo remodelling of vascularizing engineered tissues. Ann. Biomed. Eng..

[23] Crapo PM, Gilbert TW, Badylak SF (2011). An overview of tissue and whole organ decellularization processes. Biomaterials.

[24] Dahm-Kahler P, Wranning C, Lundmark C, Enskog A, Molne J (2008). Transplantation of the uterus in sheep: methodology and early reperfusion events. J. Obst. Gynaecol. Res..

[25] Darzi S, Urbankova I, Su K, White J, Lo C (2016). Tissue response to collagen containing polypropylene meshes in an ovine vaginal repair model. Acta Biomater..

[26] Darzi S, Werkmeister JA, Deane JA, Gargett CE (2016). Identification and characterization of human endometrial mesenchymal stem/stromal cells and their potential for cellular therapy. Stem Cells Transl. Med..

[27] Devauchelle B, Badet L, Lengele B, Morelon E, Testelin S (2006). First human face allograft: early report. Lancet.

[28] Diaz-Garcia C, Akhi SN, Wallin A, Pellicer A, Brannstrom M (2010). First report on fertility after allogeneic uterus transplantation. Acta Obstet. Gynecol. Scand..

[29] Ding L, Li X, Sun H, Su J, Lin N (2014). Transplantation of bone marrow mesenchymal stem cells on collagen scaffolds for the functional regeneration of injured rat uterus. Biomaterials.

[30] Dolmans MM, Marinescu C, Saussoy P, Van Langendonckt A, Amorim C, Donnez J (2010). Reimplantation of cryopreserved ovarian tissue from patients with acute lymphoblastic leukemia is potentially unsafe. Blood.

[31] Donnez J, Dolmans MM, Diaz C, Pellicer A (2015). Ovarian cortex transplantation: time to move on from experimental studies to open clinical application. Fertil. Steril..

[32] Donnez J, Dolmans MM, Pellicer A, Diaz-Garcia C, Sanchez Serrano M (2013). Restoration of ovarian activity and pregnancy after transplantation of cryopreserved ovarian tissue: a review of 60 cases of reimplantation. Fertil. Steril..

[33] Dubernard JM, Owen E, Herzberg G, Lanzetta M, Martin X (1999). Human hand allograft: report on first 6 months. Lancet.

[34] El-Akouri RR, Molne J, Groth K, Kurlberg G, Brannstrom M (2006). Rejection patterns in allogeneic uterus transplantation in the mouse. Hum. Reprod..

[35] Feinberg AW (2012). Engineered tissue grafts: opportunities and challenges in regenerative medicine. Wiley Interdiscip. Rev..

[36] Fu RH, Wang YC, Liu SP, Shih TR, Lin HL (2014). Decellularization and recellularization technologies in tissue engineering. Cell Transpl..

[37] Gargett CE, Schwab KE, Zillwood RM, Nguyen HP, Wu D (2009). Isolation and culture of epithelial progenitors and mesenchymal stem cells from human endometrium. Biol. Reprod..

[38] Godinho MJ, Teh L, Pollett MA, Goodman D, Hodgetts SI (2013). Immunohistochemical, ultrastructural and functional analysis of axonal regeneration through peripheral nerve grafts containing Schwann cells expressing BDNF, CNTF or NT3. PloS ONE.

[39] Gonfiotti A, Jaus MO, Barale D, Baiguera S, Comin C (2014). The first tissue-engineered airway transplantation: 5-year follow-up results. Lancet.

[40] Green LJ, Shikanov A (2016). In vitro culture methods of preantral follicles. Theriogenology.

[41] Hellström M, El-Akouri RR, Sihlbom C, Olsson BM, Lengqvist J (2014). Towards the development of a bioengineered uterus: comparison of different protocols for rat uterus decellularization. Acta Biomater..

[42] Hellström M, Moreno-Moya JM, Bandstein S, Bom E, Akouri RR (2016). Bioengineered uterine tissue supports pregnancy in a rat model. Fertil. Steril..

[43] Hiraoka T, Hirota Y, Saito-Fujita T, Matsuo M, Egashira M (2016). STAT3 accelerates uterine epithelial regeneration in a mouse model of decellularized uterine matrix transplantation. JCI Insight.

[44] Hoganson DM, O’Doherty EM, Owens GE, Harilal DO, Goldman SM (2010). The retention of extracellular matrix proteins and angiogenic and mitogenic cytokines in a decellularized porcine dermis. Biomaterials.

[45] House M, Sanchez CC, Rice WL, Socrate S, Kaplan DL (2010). Cervical tissue engineering using silk scaffolds and human cervical cells. Tissue Eng. A.

[46] Hu Y, Leaver SG, Plant GW, Hendriks WT, Niclou SP (2005). Lentiviral-mediated transfer of CNTF to schwann cells within reconstructed peripheral nerve grafts enhances adult retinal ganglion cell survival and axonal regeneration. Mol. Ther..

[47] Hynes RO (2009). The extracellular matrix: not just pretty fibrils. Science.

[48] Johannesson L, Enskog A, Dahm-Kahler P, Hanafy A, Chai DC (2012). Uterus transplantation in a non-human primate: long-term follow-up after autologous transplantation. Hum. Reprod..

[49] Jungebluth P, Alici E, Baiguera S, Blomberg P, Bozoky B (2011). Tracheobronchial transplantation with a stem-cell-seeded bioartificial nanocomposite: a proof-of-concept study. Lancet.

[50] Kehl D, Weber B, Hoerstrup SP (2016). Bioengineered living cardiac and venous valve replacements: current status and future prospects. Cardiovasc. Pathol..

[51] Kim MR, Park DW, Lee JH, Choi DS, Hwang KJ (2005). Progesterone-dependent release of transforming growth factor-beta1 from epithelial cells enhances the endometrial decidualization by turning on the Smad signalling in stromal cells. Mol. Hum. Reprod..

[52] Krampera M, Cosmi L, Angeli R, Pasini A, Liotta F (2006). Role for interferon-gamma in the immunomodulatory activity of human bone marrow mesenchymal stem cells. Stem Cells.

[53] Lancet E (2016). Expression of concern–Tracheobronchial transplantation with a stem-cell-seeded bioartificial nanocomposite: a proof-of-concept study. Lancet.

[54] Lane SW, Williams DA, Watt FM (2014). Modulating the stem cell niche for tissue regeneration. Nat. Biotechnol..

[55] Lang R, Stern MM, Smith L, Liu Y, Bharadwaj S (2011). Three-dimensional culture of hepatocytes on porcine liver tissue-derived extracellular matrix. Biomaterials.

[56] Lee RH, Pulin AA, Seo MJ, Kota DJ, Ylostalo J (2009). Intravenous hMSCs improve myocardial infarction in mice because cells embolized in lung are activated to secrete the anti-inflammatory protein TSG-6. Cell Stem Cell.

[57] L’Heureux N, Dusserre N, Marini A, Garrido S, de la Fuente L, McAllister T (2007). Technology insight: the evolution of tissue-engineered vascular grafts—from research to clinical practice. Nat. Clin. Pract. Cardiovasc. Med..

[58] Lichtenberg A, Tudorache I, Cebotari S, Ringes-Lichtenberg S, Sturz G (2006). In vitro re-endothelialization of detergent decellularized heart valves under simulated physiological dynamic conditions. Biomaterials.

[59] Londra L, Chuong FS, Kolp L (2015). Mayer–Rokitansky–Kuster–Hauser syndrome: a review. Int. J. Women Health.

[60] Lu SH, Wang HB, Liu H, Wang HP, Lin QX (2009). Reconstruction of engineered uterine tissues containing smooth muscle layer in collagen/matrigel scaffold in vitro. Tissue Eng. A.

[61] Luyckx V, Dolmans MM, Vanacker J, Legat C, Fortuno Moya C (2014). A new step toward the artificial ovary: survival and proliferation of isolated murine follicles after autologous transplantation in a fibrin scaffold. Fertil. Steril..

[62] Luyckx V, Dolmans MM, Vanacker J, Scalercio SR, Donnez J, Amorim CA (2013). First step in developing a 3D biodegradable fibrin scaffold for an artificial ovary. J. Ovarian Res..

[63] Ma R, Li M, Luo J, Yu H, Sun Y (2013). Structural integrity, ECM components and immunogenicity of decellularized laryngeal scaffold with preserved cartilage. Biomaterials.

[64] Macchiarini P, Jungebluth P, Go T, Asnaghi MA, Rees LE (2008). Clinical transplantation of a tissue-engineered airway. Lancet.

[65] Mao AS, Mooney DJ (2015). Regenerative medicine: current therapies and future directions. Proc. Natl. Acad. Sci. USA.

[66] Meng CX, Andersson KL, Bentin-Ley U, Gemzell-Danielsson K, Lalitkumar PG (2009). Effect of levonorgestrel and mifepristone on endometrial receptivity markers in a three-dimensional human endometrial cell culture model. Fertil. Steril..

[67] Milliez J (2009). Uterine transplantation FIGO committee for the ethical aspects of human reproduction and women’s health. Int. J. Gynaecol. Obstet..

[68] Miyazaki K, Maruyama T (2014). Partial regeneration and reconstruction of the rat uterus through recellularization of a decellularized uterine matrix. Biomaterials.

[69] Nyame TT, Chiang HA, Leavitt T, Ozambela M, Orgill DP (2015). Tissue-engineered skin substitutes. Plast. Reconstr. Surg..

[70] Olausson M, Johannesson L, Brattgard D, Diaz-Garcia C, Lundmark C (2014). Ethics of uterus transplantation with live donors. Fertil. Steril..

[71] Olausson M, Patil PB, Kuna VK, Chougule P, Hernandez N (2012). Transplantation of an allogeneic vein bioengineered with autologous stem cells: a proof-of-concept study. Lancet.

[72] Ono M, Maruyama T, Masuda H, Kajitani T, Nagashima T (2007). Side population in human uterine myometrium displays phenotypic and functional characteristics of myometrial stem cells. Proc. Natl. Acad. Sci. USA.

[73] Ott HC, Clippinger B, Conrad C, Schuetz C, Pomerantseva I (2010). Regeneration and orthotopic transplantation of a bioartificial lung. Nat. Med..

[74] Ott HC, Matthiesen TS, Goh SK, Black LD, Kren SM (2008). Perfusion-decellularized matrix: using nature’s platform to engineer a bioartificial heart. Nat. Med..

[75] Park DW, Choi DS, Ryu HS, Kwon HC, Joo H, Min CK (2003). A well-defined in vitro three-dimensional culture of human endometrium and its applicability to endometrial cancer invasion. Cancer Lett..

[76] Peloso A, Dhal A, Zambon JP, Li P, Orlando G (2015). Current achievements and future perspectives in whole-organ bioengineering. Stem Cell Res. Ther..

[77] Petersen TH, Calle EA, Zhao L, Lee EJ, Gui L (2010). Tissue-engineered lungs for in vivo implantation. Science.

[78] Racho El-Akouri R, Kurlberg G, Brannstrom M (2003). Successful uterine transplantation in the mouse: pregnancy and post-natal development of offspring. Hum. Reprod..

[79] Racho El-Akouri R, Kurlberg G, Dindelegan G, Molne J, Wallin A, Brannstrom M (2002). Heterotopic uterine transplantation by vascular anastomosis in the mouse. J. Endocrinol..

[80] Raya-Rivera AM, Esquiliano D, Fierro-Pastrana R, Lopez-Bayghen E, Valencia P (2014). Tissue-engineered autologous vaginal organs in patients: a pilot cohort study. Lancet.

[81] Reing JE, Zhang L, Myers-Irvin J, Cordero KE, Freytes DO (2009). Degradation products of extracellular matrix affect cell migration and proliferation. Tissue Eng. A.

[82] Sadri-Ardekani H, Atala A (2015). Regenerative medicine for the treatment of reproductive system disorders: current and potential options. Adv. Drug Deliv. Rev..

[83] San Juan F, Cortes M (2011). Mortality on the waiting list for liver transplantation: management and prioritization criteria. Transpl. Proc..

[84] Santoso EG, Yoshida K, Hirota Y, Aizawa M, Yoshino O (2014). Application of detergents or high hydrostatic pressure as decellularization processes in uterine tissues and their subsequent effects on in vivo uterine regeneration in murine models. PloS ONE.

[85] Sayegh MH, Carpenter CB (2004). Transplantation 50 years later–progress, challenges, and promises. N Engl. J. Med..

[86] Scarritt ME, Pashos NC, Bunnell BA (2015). A review of cellularization strategies for tissue engineering of whole organs. Front. Bioeng. Biotechnol..

[87] Schutte SC, Taylor RN (2012). A tissue-engineered human endometrial stroma that responds to cues for secretory differentiation, decidualization, and menstruation. Fertil. Steril..

[88] Sengupta S, Sengupta J, Mittal S, Kumar S, Ghoshi D (2008). Effect of human chorionic gonadotropin (hCG) on expression of vascular endothelial growth factor a (VEGF-a) in human mid-secretory endometrial cells in three-dimensional primary culture. Indian J. Physiol. Pharmacol..

[89] Shea LD, Woodruff TK, Shikanov A (2014). Bioengineering the ovarian follicle microenvironment. Ann. Rev. Biomed. Eng..

[90] Shikanov A, Smith RM, Xu M, Woodruff TK, Shea LD (2011). Hydrogel network design using multifunctional macromers to coordinate tissue maturation in ovarian follicle culture. Biomaterials.

[91] Shikanov A, Xu M, Woodruff TK, Shea LD (2009). Interpenetrating fibrin-alginate matrices for in vitro ovarian follicle development. Biomaterials.

[92] Song JJ, Guyette JP, Gilpin SE, Gonzalez G, Vacanti JP, Ott HC (2013). Regeneration and experimental orthotopic transplantation of a bioengineered kidney. Nat. Med..

[93] Stanton AL, Lobel M, Sears S, DeLuca RS (2002). Psychosocial aspects of selected issues in women’s reproductive health: current status and future directions. J. Consult. Clin. Psychol..

[94] Tang D, Tare RS, Yang LY, Williams DF, Ou KL, Oreffo RO (2016). Biofabrication of bone tissue: approaches, challenges and translation for bone regeneration. Biomaterials.

[95] Totonelli G, Maghsoudlou P, Garriboli M, Riegler J, Orlando G (2012). A rat decellularized small bowel scaffold that preserves villus-crypt architecture for intestinal regeneration. Biomaterials.

[96] Trappmann B, Gautrot JE, Connelly JT, Strange DG, Li Y (2012). Extracellular-matrix tethering regulates stem-cell fate. Nat. Mater..

[97] Ulrich D, Edwards SL, Alexander DL, Rosamilia A, Werkmeister JA (2016). Changes in pelvic organ prolapse mesh mechanical properties following implantation in rats. Am. J. Obstet. Gynecol..

[98] Ulrich D, Edwards SL, Su K, Tan KS, White JF (2014). Human endometrial mesenchymal stem cells modulate the tissue response and mechanical behavior of polyamide mesh implants for pelvic organ prolapse repair. Tissue Eng. A.

[99] Ulrich D, Muralitharan R, Gargett CE (2013). Toward the use of endometrial and menstrual blood mesenchymal stem cells for cell-based therapies. Expert Opin. Biol. Ther..

[100] Uygun BE, Soto-Gutierrez A, Yagi H, Izamis ML, Guzzardi MA (2010). Organ reengineering through development of a transplantable recellularized liver graft using decellularized liver matrix. Nat. Med..

[101] Vanacker J, Dolmans MM, Luyckx V, Donnez J, Amorim CA (2014). First transplantation of isolated murine follicles in alginate. Regen. Med..

[102] Vanacker J, Luyckx V, Dolmans MM, Des Rieux A, Jaeger J (2012). Transplantation of an alginate-matrigel matrix containing isolated ovarian cells: first step in developing a biodegradable scaffold to transplant isolated preantral follicles and ovarian cells. Biomaterials.

[103] Wang L, Johnson JA, Chang DW, Zhang Q (2013). Decellularized musculofascial extracellular matrix for tissue engineering. Biomaterials.

[104] Wang HB, Lu SH, Lin QX, Feng LX, Li DX (2010). Reconstruction of endometrium in vitro via rabbit uterine endometrial cells expanded by sex steroid. Fertil. Steril..

[105] Watson CJ, Dark JH (2012). Organ transplantation: historical perspective and current practice. Br. J. Anaesth..

[106] Watt FM, Huck WT (2013). Role of the extracellular matrix in regulating stem cell fate. Nat. Rev. Mol. Cell Biol..

[107] West ER, Shea LD, Woodruff TK (2007). Engineering the follicle microenvironment. Sem. Reprod. Med..

[108] Wong ML, Wong JL, Vapniarsky N, Griffiths LG (2016). In vivo xenogeneic scaffold fate is determined by residual antigenicity and extracellular matrix preservation. Biomaterials.

[109] Wranning CA, Akhi SN, Diaz-Garcia C, Brannstrom M (2011). Pregnancy after syngeneic uterus transplantation and spontaneous mating in the rat. Hum. Reprod..

[110] Wranning CA, El-Akouri RR, Lundmark C, Dahm-Kahler P, Molne J (2006). Auto-transplantation of the uterus in the domestic pig (Sus scrofa): Surgical technique and early reperfusion events. J. Obstet. Gynaecol. Res..

[111] Wranning CA, Marcickiewicz J, Enskog A, Dahm-Kahler P, Hanafy A, Brannstrom M (2010). Fertility after autologous ovine uterine-tubal-ovarian transplantation by vascular anastomosis to the external iliac vessels. Hum. Reprod..

[112] Xu M, Kreeger PK, Shea LD, Woodruff TK (2006). Tissue-engineered follicles produce live, fertile offspring. Tissue Eng..

[113] Young RC, Goloman G (2013). Allo- and xeno-reassembly of human and rat myometrium from cells and scaffolds. Tissue Eng. A.

